# Common Seasonal Pathogens and Epidemiology of Henoch-Schönlein Purpura Among Children

**DOI:** 10.1001/jamanetworkopen.2024.5362

**Published:** 2024-04-05

**Authors:** Arthur Felix, Zein Assad, Philippe Bidet, Marion Caseris, Cécile Dumaine, Albert Faye, Isabelle Melki, Florentia Kaguelidou, Zaba Valtuille, Naïm Ouldali, Ulrich Meinzer

**Affiliations:** 1 Pediatric Internal Medicine, Rheumatology and Infectious Diseases, National Reference Centre for Rare Pediatric Inflammatory Rheumatisms and Systemic Autoimmune Diseases (RAISE), Department of General Pediatrics, Robert-Debré University Hospital, Assistance Publique-Hôpitaux de Paris, Paris, France; 2 Competence Centre RAISE Antilles-Guyane, EpiCliV Research Unit, Department of General Pediatrics, Martinique University Hospital, University of French West Indies, Martinique, France; 3 Infection, Antimicrobials, Modeling, Evolution, Paris Cité University, INSERM UMR 1137, Paris, France; 4 Department of Microbiology, Robert-Debré University Hospital, Assistance Publique-Hôpitaux de Paris, Paris, France; 5 Université Paris Cité, INSERM UMR-1123, ECEVE, Paris, France; 6 Pediatrics, Rheumatology and Pediatric Internal Medicine, Children’s Hospital, Bordeaux, France; 7 Center of Clinical Investigations, INSERM CIC1426, Robert-Debré University Hospital, Assistance Publique-Hôpitaux de Paris, Paris, France; 8 Centre de Recherche sur l’inflammation UMR 1149, Université Paris Cité, INSERM, Paris, France

## Abstract

**Importance:**

Henoch-Schönlein purpura (HSP) is the most common type of vasculitis in children. The factors that trigger the disease are poorly understood. Although several viruses and seasonal bacterial infections have been associated with HSP, differentiating the specific associations of these pathogens with the onset of HSP remains a challenge due to their overlapping seasonal patterns.

**Objective:**

To analyze the role of seasonal pathogens in the epidemiology of HSP.

**Design, Setting, and Participants:**

This cohort study comprised an interrupted time-series analysis of patient records from a comprehensive national hospital-based surveillance system. Children younger than 18 years hospitalized for HSP in France between January 1, 2015, and March 31, 2023, were included.

**Exposure:**

Implementation and relaxation of nonpharmaceutical interventions (NPIs) for the COVID-19 pandemic, such as social distancing and mask wearing.

**Main Outcomes and Measures:**

The main outcomes were the monthly incidence of HSP per 100 000 children, analyzed via a quasi-Poisson regression model, and the estimated percentage of HSP incidence potentially associated with 14 selected common seasonal pathogens over the same period.

**Results:**

The study included 9790 children with HSP (median age, 5 years [IQR, 4-8 years]; 5538 boys [56.4%]) and 757 110 children with the infectious diseases included in the study (median age, 0.7 years [IQR, 0.2-2 years]; 393 697 boys [52.0%]). The incidence of HSP decreased significantly after implementation of NPIs in March 2020 (−53.6%; 95% CI, −66.6% to −40.6%; *P* < .001) and increased significantly after the relaxation of NPIs in April 2021 (37.2%; 95% CI, 28.0%-46.3%; *P* < .001). The percentage of HSP incidence potentially associated with *Streptococcus pneumoniae* was 37.3% (95% CI, 22.3%-52.3%; *P* < .001), the percentage of cases associated with *Streptococcus pyogenes* was 25.6% (95% CI, 16.7%-34.4%; *P* < .001), and the percentage of cases associated with human rhino enterovirus was 17.1% (95% CI, 3.8%-30.4%; *P* = .01). Three sensitivity analyses found similar results.

**Conclusions and Relevance:**

This study found that significant changes in the incidence of HSP simultaneously with major shifts in circulating pathogens after NPIs for the COVID-19 pandemic indicated that approximately 60% of HSP incidence was potentially associated with pneumococcus and group A streptococcus. This finding suggests that preventive measures against these pathogens could reduce the incidence of pediatric HSP.

## Key Points

**Question:**

What are the associations of the main seasonal pathogens with the epidemiology of Henoch-Schönlein purpura (HSP)?

**Findings:**

In this cohort study of 9790 children with HSP and 757 110 children with an infectious disease, time-series analysis of a prospective national surveillance cohort from 2015 to 2023 revealed that 37.3% of HSP incidence was potentially associated with *Streptococcus pneumoniae* and that 25.6% of HSP incidence was potentially associated with *Streptococcus pyogenes*. In contrast, all other seasonal pathogens played a minor role.

**Meaning:**

These findings underscore the potentially significant role of *S pneumoniae* and *S pyogenes* in the burden of childhood HSP, suggesting that preventive measures could prove effective for this common form of childhood vasculitis.

## Teaser

This cohort study analyzes the role of seasonal pathogens in the epidemiology of Henoch-Schönlein purpura among children in France before, during, and after the COVID-19 pandemic.

## Introduction

Henoch-Schönlein purpura (HSP), also known as immunoglobulin A (IgA) vasculitis in adults, is the most common type of vasculitis in children, affecting mainly small blood vessels and caused by the deposition of immune complexes.^1^ It typically affects the skin and less frequently the digestive system, joints, and kidneys.^2^ The exact cause and underlying mechanisms of HSP are not fully understood, but it is believed to result from an abnormal inflammatory response triggered by antigenic stimuli, particularly infectious agents, in individuals with a genetic predisposition.

Henoch-Schönlein purpura is more frequent during the autumn and winter months in Western countries and is often preceded by an upper respiratory tract infection.^2^ Several seasonal viruses and bacterial infections have been associated with childhood HSP.^3^ However, differentiating the specific associations of these pathogens with the onset of HSP remains a challenge due to their overlapping seasonal patterns. The SARS-CoV-2 pandemic led to a marked reduction in the circulation of infectious pathogens in the pediatric population as a result of international nonpharmaceutical interventions (NPIs), such as social distancing and mask wearing.^4,5,6^ Subsequently, after these NPIs were relaxed in the spring of 2021, unusual outbreaks of viral and bacterial pathogens were reported in France and other countries.^7,8,9^ Given the suspected role of seasonal pathogens in the pathophysiology of HSP, we hypothesized that these unprecedented changes in the circulation of seasonal pathogens after the implementation and then relaxation of NPIs would represent a unique opportunity to better identify the role of individual seasonal pathogens in the epidemiology of HSP. In this study, our primary goals were to investigate the association of SARS-CoV-2 pandemic–related NPIs with the incidence of HSP among children and to assess the proportions of HSP cases that might be associated with specific seasonal viral and bacterial pathogens.

## Methods

### Study Design

We conducted a population-based interrupted time-series analysis of patient data from a hospital-based French national surveillance system from January 1, 2015, to March 31, 2023. Access to the French Medicalization of Information Systems Program (Programme de Médicalisation des Systèmes d'Information [PMSI]) was requested from and approved by the National Commission on Information and Liberty. As part of an ongoing continuous mission of public health using anonymous aggregated data for public health purposes, this study did not require ethical committee approval or written informed consent, based on the 2021 National Data Protection Act. The Strengthening the Reporting of Observational Studies in Epidemiology (STROBE) reporting guideline was followed to report this study.^10^

### Study Data and Settings

The data were obtained from the PMSI, which is a comprehensive national database that contains all hospital discharge records in France, as previously described.^11^ Diagnoses related to the hospitalizations were recorded according to the *International Statistical Classification of Diseases and Related Health Problems, Tenth Revision* (*ICD-10*).

### Children Hospitalized for HSP

We included all children younger than 18 years hospitalized for HSP in France between January 1, 2015, and March 31, 2023. Henoch-Schönlein purpura was defined as *ICD-10* code D690. We excluded other types of pediatric purpura, such as qualitative platelet defects (code D691), immune thrombocytopenic purpura (code D693), hypersensitivity angiitis (code M310), thrombotic microangiopathy (code M311), Waterhouse-Friderichsen syndrome (code A391), disseminated intravascular coagulation (code D65), neonatal cutaneous hemorrhage (code P545), other nonthrombocytopenic purpura (code D692), and primary thrombocytopenia (code D694). The following data were recorded for each inpatient stay: age, sex, date, length of hospital stay, and death. For children with multiple hospital stays for HSP, we considered only the first hospital stay as the date of diagnosis.

### Nationwide Circulation of Pathogens

We also recorded all hospitalized children younger than 18 years with an infection due to the following pathogens: human rhino enterovirus (hRV-hEV), influenza, respiratory syncytial virus, human parainfluenza virus, human metapneumovirus, human adenovirus, non–SARS-CoV-2 human coronavirus, varicella zoster virus, norovirus, rotavirus, *Streptococcus pneumoniae*, *Streptococcus pyogenes*, *Mycoplasma pneumoniae*, and *Chlamydia pneumoniae*. The details of *ICD-10* codes are presented in eTable 1 in Supplement 1. Infections were identified using polymerase chain reaction (PCR), a rapid antigen detection test, or bacterial culture in hospital laboratories. There was no change in the national guidelines for the detection of these pathogens during the study period. Because hRV and hEV are difficult to distinguish by most routine multiplex reverse transcription (RT)–PCR assays, because of their RNA sequence similarities, we chose to consider them as 1 entity (hRV-hEV).^12^ Data were aggregated into a monthly level to calculate the monthly incidence of HSP and selected pathogens per 100 000 children. We used the age-specific French population demographics, obtained from the National Institute of Statistics and Economic Studies,^13^ as the denominator.

### Study Periods

We organized the study into 3 periods according to the NPIs applied in France to counter the SARS-CoV-2 pandemic, as previously published.^14^ The pre-NPI period was from January 2015 to March 2020, the NPI period was from April 2020 to March 2021, and the lifting of NPIs was from April 2021 to March 2023.

### Outcomes and Measures

The main outcomes were the monthly incidence of HSP per 100 000 children in France and the estimated percentage of HSP incidence associated with the selected pathogens. Secondary outcomes were the monthly incidence of HSP by age groups (≤5 years, 6-10 years, and 11-17 years). As a control outcome, we analyzed the monthly incidence of acute pyelonephritis per 100 000 children over the same period to assess the risk of bias due to potential hidden cointerventions, mainly a potential change in hospital admission capacities during the SARS-CoV-2 pandemic.^14,15^

### Statistical Analysis

First, we built a quasi-Poisson regression model to estimate the changes in the incidence HSP after the implementation and relaxation of NPIs. This model accounted for temporal trends before these interventions and for the seasonal pattern of HSP using harmonic terms (sines and cosines with 6- and 12-month periods). The time unit set was 1 month.^16^ The final model was used to produce a counterfactual estimation that assumed the NPIs were not implemented, based on disease data from the 5 pre-NPI years. We included step and slope variables as regressors in the model to estimate an immediate change in the incidence of HSP after the implementation of NPIs in March 2020^6,17^ and a progressive change after their lifting, starting in April 2021, assuming that the relaxation of the containment measures was gradual. Second, we calculated the percentage of HSP incidence associated with each pathogen of interest. The term *potentially attributable fraction* is an epidemiologic term reflecting a temporal association between the incidence of HSP and the population-level circulation of studied pathogens. Thus, we fitted the incidence of HSP using the seasonally adjusted (3-, 6-, and 12-month periods) quasi-Poisson model including all pathogens as explanatory variables. Then, we estimated the expected incidence of HSP if each pathogen was not present during the study period by using the same equation and set each pathogen term equal to zero. The estimated percentage of HSP incidence associated with each pathogen was calculated as the relative change in the incidence of HSP that would occur over the whole study period if the exposure to each pathogen was removed (eMethods in Supplement 1).^11,18^

We performed 4 sensitivity analyses to assess the robustness of the study findings: (1) a quasi-Poisson model with 6- and 12-month period seasonality; (2) a trigonometric quasi-Poisson regression with the monthly counts of the outcomes of interest instead of incidences; (3) a quasi-Poisson model excluding highly correlated covariates that could affect the multiple regression model; and (4) a model calculating the percentage of HSP cases associated with SARS-CoV-2. This multicollinearity was assessed using the generalized variance inflation factor.^19^ Statistical tests were 2-sided, with *P* < .05 considered statistically significant. The validity of the models was assessed by visual inspection of the correlograms and analysis of the residuals. Analyses were performed using R software, version 4.3.1 (R Project for Statistical Computing).

## Results

We included 9790 children (median age, 5 years [IQR, 4-8 years]; 5538 boys [56.4%]) hospitalized with a diagnosis of HSP between January 2015 and March 2023. Patient characteristics are presented in Table 1.

**Table 1.** Baseline Characteristics of Hospitalizations of Children With Henoch-Schönlein Purpura, January 2015 to March 2023

Characteristic	Before NPIs (n = 7473)^a^	NPI implementation (n = 592)^b^	NPI lifting (n = 1725)^c^	Total (N = 9790)
Age, median (IQR), y	5 (4-8)	5 (4-8)	5 (4-8)	5 (4-8)
Sex, No. (%)				
Male	4213 (56.4)	341 (57.6)	984 (57.0)	5538 (56.6)
Female	3260 (43.6)	251 (42.4)	741 (43.0)	4252 (43.4)
Length of stay, median (IQR), d	2 (1-3)	2 (1-3)	2 (1-3)	2 (1-3)
Hospital deaths, No. (%)	5 (0.07)	1 (0.2)	0	6 (0.06)

Abbreviation: NPI, nonpharmaceutical intervention.

^a^ From January 2015 to March 2020.

^b^ From April 2020 to March 2021.

^c^ From April 2021 to March 2023.

**Figure 1.**
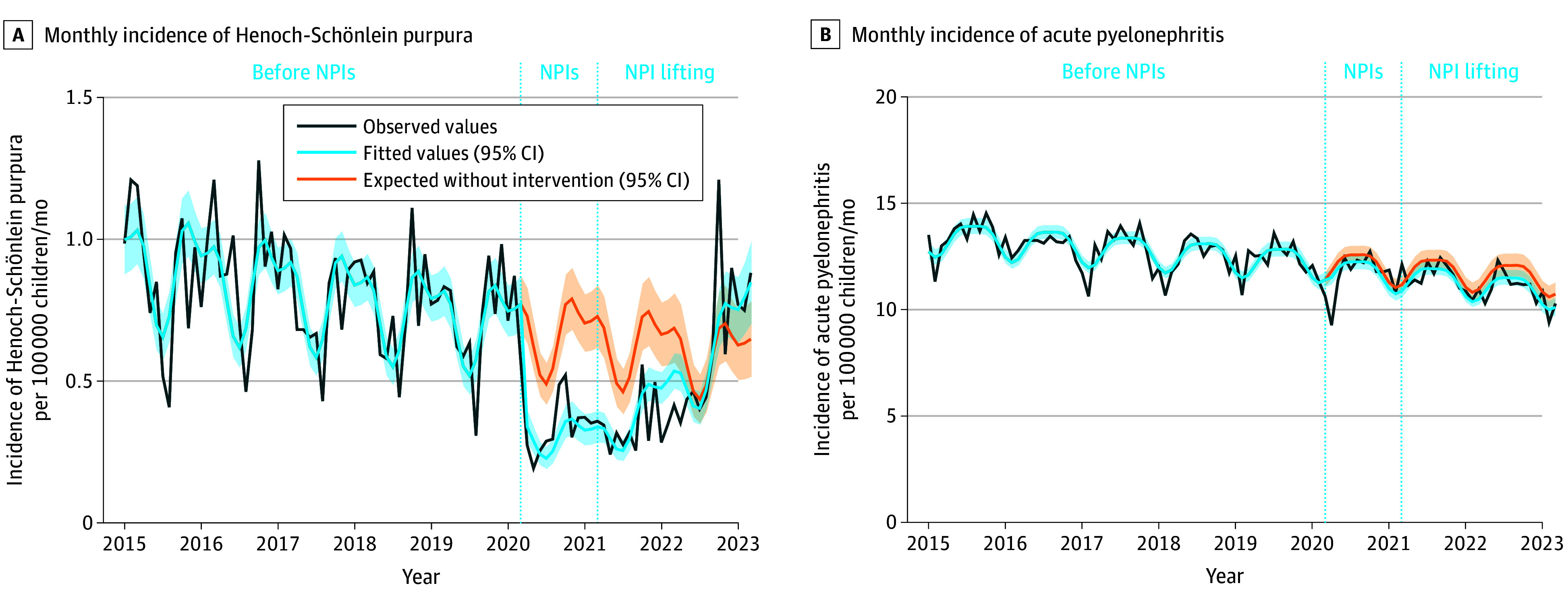
Association of Nonpharmaceutical Intervention (NPI) Implementation and Lifting With the Monthly Incidence of Henoch-Schönlein Purpura and Acute Pyelonephritis per 100 000 Children Younger Than 18 Years in France, January 2015 to March 2023 A, Monthly incidence of Henoch-Schönlein purpura per 100 000 children younger than 18 years (n = 9790). B, Monthly incidence of acute pyelonephritis per 100 000 children younger than 18 years (n = 177 791). The dark blue line shows the observed data. The light blue line shows the model estimates based on observed data using the quasi-Poisson regression model. The orange line shows the expected values without NPI implementation using the same quasi-Poisson model. The light blue and orange shaded areas indicate the 95% CIs. The dotted vertical lines indicate the implementation of the NPIs in March 2020 and the lifting of NPIs in April 2021.

We found a significant decrease in the incidence of HSP associated with the implementation of the SARS-CoV-2–related NPIs in March 2020 (immediate change, −53.6%; 95% CI, −66.6% to −40.6%; *P* < .001), followed by a significant increase after the lifting of the NPIs since April 2021 (progressive change, 37.2%; 95% CI, 28.0%-46.3%; *P* < .001) (Figure 1A and Table 2). These results were similar for all age groups (≤5, 6-10, and 11-17 years). In comparison, the monthly incidence of acute pyelonephritis per 100 000 children (NPI implementation: –2.6; 95% CI, –6.2 to 1.0; NPI lifting: –1.6; 95% CI, –4.2 to 1.0) was stable, suggesting that the national surveillance system or hospital admission capacities did not change over the study period (Figure 1B and Table 2).

**Table 2.** Association of the Implementation and Lifting of NPIs With the Monthly Incidence of HSP per 100 000 Children Younger Than 18 Years in France, January 2015 to March 2023

Outcome	NPI implementation^a^		NPI lifting^b^	
	Immediate change, % (95% CI)	*P* value for immediate change	Progressive change, % (95% CI)	*P* value for progressive change
Monthly incidence of HSP per 100 000 children^c^	−53.6 (−66.6 to −40.6)	<.001	37.2 (28.0 to 46.3)	<.001
Monthly incidence of HSP per 100 000 children by age group, y^c^				
≤5	−46.3 (−62.7 to −29.9)	<.001	35.7 (23.9 to 47.6)	<.001
6-10	−61.4 (−75.7 to −47.2)	<.001	42.0 (32.2 to 47.0)	<.001
11-17	−41.1 (−61.1 to −21.0)	<.001	24.1 (9.9 to 31.3)	.001
Control outcome^c^				
Monthly incidence of acute pyelonephritis per 100 000 children	−2.6 (−6.2 to 1.0)	.16	−1.6 (−4.2 to 1.0)	.22

Abbreviations: HSP, Henoch-Schönlein purpura; NPI, nonpharmaceutical intervention.

^a^ NPI implementation was in April 2020.

^b^ Lifting of NPIs started in April 2021.

^c^ Analysis by seasonally adjusted quasi-Poisson regression model.

**Figure 2.**
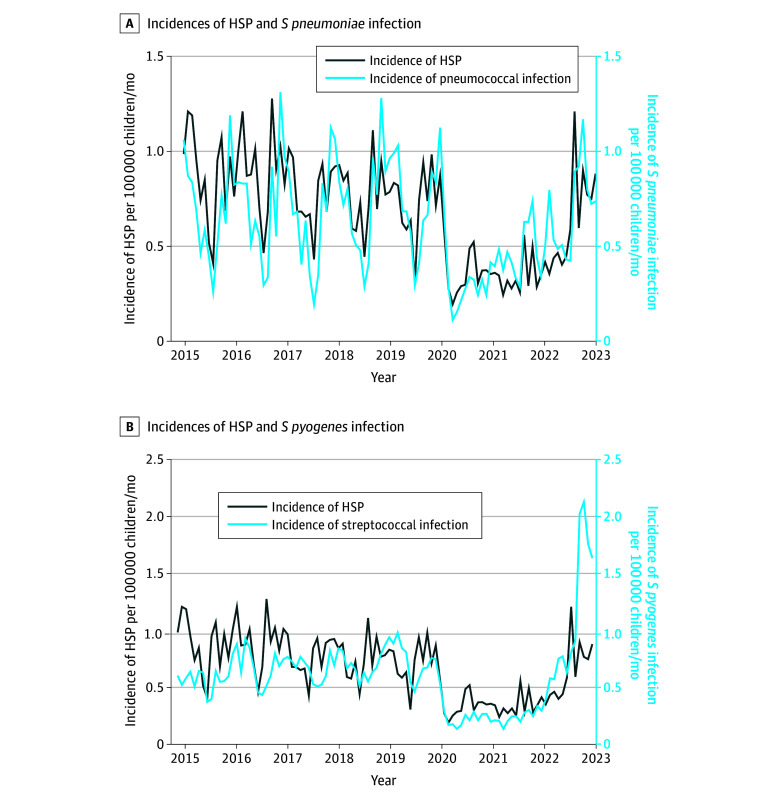
Seasonal Pattern of the Incidences of Henoch-Schönlein Purpura (HSP) and *Streptococcus pneumoniae* and of HSP and *Streptococcus pyogenes* Infections per 100 000 Children Younger Than 18 Years in France A, Incidences of HSP and *S pneumoniae* infections (n = 9037) per 100 000 children younger than 18 years. B, Incidences of HSP and *S pyogenes* infections (n = 8576) per 100 000 children younger than 18 years. The dark blue line shows the monthly incidence of HSP per 100 000 children.

During the same period, 757 110 children (median age, 0.7 years [IQR, 0.2-2 years]; 393 697 boys [52.0%]) with the selected infectious diseases were included. The characteristics of patients hospitalized for each infectious disease are shown in eTable 2 in Supplement 1. The course of HSP showed a seasonal pattern, with a higher incidence from September to December, which was similar to the seasonality of *S pneumoniae* and *S pyogenes* (Figure 2). Using the multivariate quasi-Poisson regression model, the percentage of HSP incidence potentially associated with *S pneumoniae* was 37.3% (95% CI, 22.3%-52.3%; *P* < .001), the percentage associated with *S pyogenes* was 25.6% (95% CI, 16.7%-34.4*%; P* < .001), and the percentage associated with hRV-hEV was 17.1% (95% CI, 3.8%-30.4%; *P* = .01) (Table 3). No association was found for the other tested pathogens, including SARS-CoV-2 (eTable 3 and eFigure 1 in Supplement 1). The quality assessment of the models was satisfactory (eFigure 2 in Supplement 1). The 3 sensitivity analyses showed similar results (eTable 4 in Supplement 1).

**Table 3.** Estimated Percentage of HSP Potentially Associated With Seasonal Pathogens

Pathogen	HSP incidence^a^	
	Estimated % (95% CI)	*P* value
Virus		
hRV-hEV	17.1 (3.8 to 30.4)	.01
Influenza virus	0.54 (−3.6 to 4.7)	.80
RSV	−6.9 (−14.4 to 0.60)	.08
hPIV	8.2 (−0.76 to 17.2)	.08
hMPV	−7.2 (−15.6 to 1.1)	.09
hAdV	−7.8 (−27.0 to 11.4)	.43
hCoV	1.7 (−7.9 to 11.4)	.73
VZV	−2.6 (−17.1 to 12.0)	.73
Norovirus	−3.0 (−11.8 to 5.7)	.50
Rotavirus	5.9 (−2.7 to 14.4)	.18
Bacteria		
* Streptococcus pneumoniae*	37.3 (22.3 to 52.3)	<.001
* Streptococcus pyogenes*	25.6 (16.7 to 34.4)	<.001
* Mycoplasma pneumoniae*	7.6 (−4.9 to 20.1)	.24
* Chlamydia pneumoniae*	1.0 (−8.3 to 10.3)	.83

Abbreviations: hAdV, human adenovirus; hCoV, human coronavirus; hMPV, human metapneumovirus; hPIV, human parainfluenza virus; hRV-hEV, human rhino enterovirus; HSP, Henoch-Schönlein purpura; RSV, respiratory syncytial virus; VZV, varicella zoster virus.

^a^ Analysis by seasonally adjusted quasi-Poisson regression model.

## Discussion

To our knowledge, this study is the first to reveal the shifts in HSP incidence after the COVID-19 pandemic–related NPIs, demonstrating the key role of seasonal infections in HSP epidemiology, and the temporal association of HSP incidence with outbreaks of *S pneumoniae* and *S pyogenes*. Indeed, our data demonstrated that approximately 60% of HSP incidence are potentially associated with pneumococcus and group A streptococcus. The seasonal pattern in HSP epidemiology is in line with previous data showing a predominant incidence during autumn and winter among European and Asian pediatric cohorts^20,21,22^ and among cohorts of adults with HSP or IgA nephropathy.^23^ However, before the COVID-19 pandemic, epidemiologic studies were unable to accurately identify the differential role of seasonal pathogens in HSP because of the considerable temporal overlap between them. In contrast, this study covered the SARS-CoV-2 pandemic period, when seasonal pathogens suddenly and simultaneously stopped circulating, before reemerging successively after the lifting of NPIs. In parallel, the incidence of HSP decreased significantly after the implementation of NPIs and increased progressively after the gradual removing of NPIs, when seasonal pathogens reemerged in the population.^4,5,6^ This unique experimental scenario enabled us to quantify the involvement of seasonal pathogens in triggering pediatric HSP, using the strength of a time-series analysis on a 9790-patient cohort.

The present study showed a temporal association between the incidence of HSP and the circulation of *S pneumoniae* and *S pyogenes*, highlighting the crucial role of streptococcal species in the pathophysiology of HSP. Several points merit discussion. First, previous studies carried out in several geographically disparate populations reinforce the hypothesis of a significant streptococcal role in the development of HSP. In a Chinese study including 1200 patients with HSP, positive streptococcal antibody titers in serum were present in 17% of cases.^24^ A Spanish case series also reported β-hemolytic streptococcal carriage confirmed by throat swab culture in 36% of patients with HSP.^25^ Second, positive antibody titers or nasopharyngeal cultures do not necessarily indicate an acute streptococcal infection but may also reflect carriage, which is relatively common among young children.^26,27^ This aspect underlines the main limitation of microbiological studies, which may not be adapted to reliably assess the role of streptococci in HSP. In this context, an epidemiologic study found a temporal correlation between HSP and hospital admissions for invasive group A streptococcus infections.^28^ Third, although the role of β-hemolytic streptococci in triggering HSP was suggested by previous studies,^24,28,29^ to our knowledge, there are currently no data on pneumococcal involvement in the disease. The main difficulty in establishing the association between HSP and *S pneumoniae* is that noninvasive pneumococcal diseases, which represent the highest burden of pneumococcal infections—the tip of the iceberg—are undocumented.^30^ Therefore, the population-based approach seems to represent a reliable method to estimate the potentially attributable fraction of *S pneumoniae* in HSP. In this context, this study uncovers that the spectrum of streptococcal species associated with the development of HSP extends beyond *S pyogenes* and β-hemolytic streptococci, with *S pneumoniae* unexpectedly identified as the main seasonal pathogen. Further studies are needed to assess whether specific pneumococcal serotypes may be particularly involved in triggering HSP, which would estimate the potential benefit associated with vaccine prevention and pneumococcal boosters in the general pediatric population.

Although this study demonstrated a temporal association of HSP incidence with the epidemiology of *S pneumoniae* and *S pyogenes*, it was not designed to elucidate the pathophysiological mechanisms through which streptococcal infections may trigger HSP. However, *S pneumoniae* is known for its ability to induce vasculitis-like conditions after invasive infections, with vascular complications most often occurring rapidly, a mean of 7 days after diagnosis.^31^ Although extrapolation to the context of HSP must be undertaken with great caution, these observations would be consistent with a potentially fairly direct triggering of vasculitis-relevant immune responses by streptococci. Studies analyzing patient biopsies indicate a possible mechanistic role of streptococcal surface proteins or streptococcal antigens in HSP and IgA nephropathy, as deposits of IgA-binding streptococcal surface proteins were detected in 54% of kidneys and 80% of skin biopsies^32^ and streptococcal antigen overexpression was reported in 30% of patients’ kidney mesangium.^33^ Further studies are needed to understand the exact pathologic mechanisms linking streptococcal infections to the development of HSP.

Viral infections have been considered as a possible trigger of HSP, with a long list of candidate viral agents proposed in the literature.^2,3,20,24^ Although we considered 10 of these agents in our study, we found that only hRV-hEV was associated with HSP, with a calculated attributable fraction of 17.1%. Unfortunately, our data did not allow us to further separate hEV from hRV, as the RT-PCR primers of the virus detection kits used in French hospitals detect both rhinoviruses and enteroviruses. These data suggest that viral infections are unlikely to significantly trigger HSP, unlike streptococcal infections.

### Limitations

Our study had several limitations. First, in this hospital-based surveillance system, we included patients with HSP requiring hospitalization who were likely to present with more severe forms of HSP. Thus, our results may not be extrapolated to patients with milder forms of HSP who receive only ambulatory care. In addition, information on the disease severity and organ involvement (ie, joint, intestinal, and kidney disease), which might be associated with specific pathogens, was not provided by this surveillance database. Second, the term *potentially attributable fraction* is an epidemiologic term reflecting a temporal association between the incidence of HSP and the population-level circulation of studied pathogens, which is not synonymous with a causal relationship. Third, in this study, we focused on frequent infectious agents with seasonal patterns and selected those most likely to trigger HSP on the basis of published data.^2,3,20,24,28,29,34^ We cannot exclude that other infectious agents may also be associated with HSP epidemiology. Fourth, our study methods allowed us to establish a correlation between HSP and pathogens with a precision limited to 1 month. Fifth, we were unable to enrich our epidemiologic results with disease severity parameters because the PMSI is an epidemiologic register that does not allow detailed individual clinical and biological data to be obtained. Sixth, the present study was performed in a pediatric population from a single European country, and the results should therefore be confirmed in other populations and other countries.

## Conclusions

In this cohort study, time-series analysis of a prospective national cohort study revealed shifts in the incidence of HSP after the implementation and discontinuation of COVID-19–related NPIs, concomitant with major disruptions in population-level circulation of seasonal pathogens. In addition to confirming the association between infections and HSP epidemiology, this epidemiologic scenario suggested a key role of pneumococcus and group A streptococcus in pediatric HSP epidemiology. Further studies are needed to assess the potential effect of preventing streptococcal infections on reducing the incidence of HSP.

## Notes

Supplement 1. **eTable 1.** *ICD-10* Codes for Seasonal Pathogens in the General Population **eTable 2.** Baseline Characteristics of Hospitalized Children Aged <18 Years With Infection, January 2015 to March 2023 **eTable 3.** Sensitivity Analysis With Estimated Fraction of Henoch-Schönlein Purpura Potentially Attributable to SARS-CoV-2 **eFigure 1.** Seasonal Pattern of the Incidence of HSP and SARS-CoV-2 Infections (N=41,484) per 100,000 Children Aged <18 Years in France **eFigure 2.** Correlograms and Residuals Analysis of the Final Quasi-Poisson Regression Model for the Estimated Fraction of ACS Attributable to Seasonal Pathogens **eTable 4.** Sensitivity Analyses for the Estimated Fraction of HSP Potentially Attributable to Seasonal Pathogens **eMethods.** Details of the Quasi-Poisson Regression Model

Supplement 2.
Data Sharing Statement
