# Supplementary material for: Common Seasonal Pathogens and Epidemiology of Henoch-Schönlein Purpura Among Children
Source: JAMA Netw Open. 2024 Apr 5;7(4):e245362. doi: 10.1001/jamanetworkopen.2024.5362 (PMC10998156; doi:10.1001/jamanetworkopen.2024.5362)
Supplement: Supplement 1. — eTable 1. ICD-10 Codes for Seasonal Pathogens in the General Population eTable 2. Baseline Characteristics of Hospitalized Children Aged <18 Years With Infection, January 2015 to March 2023 eTable 3. Sensitivity Analysis With Estimated Fraction of Henoch-Schönlein Purpura Potentially Attributable to SARS-CoV-2 eFigure 1. Seasonal Pattern of the Incidence of HSP and SARS-CoV-2 Infections (N=41,484) per 100,000 Children Aged <18 Years in France eFigure 2. Correlograms and Residuals Analysis of the Final Quasi-Poisson Regression Model for the Estimated Fraction of ACS Attributable to Seasonal Pathogens eTable 4. Sensitivity Analyses for the Estimated Fraction of HSP Potentially Attributable to Seasonal Pathogens eMethods. Details of the Quasi-Poisson Regression Model [file jamanetwopen-e245362-s001.pdf]

Supplementary Online Content

Felix A, Assad Z, Bidet P, et al. Common seasonal pathogens and epidemiology of Henoch-Schönlein purpura among children. *JAMA Netw Open.* 2024;7(4):e245362. doi:10.1001/jamanetworkopen.2024.5362

eTable 1. *ICD-10* Codes for Seasonal Pathogens in the General Population
eTable 2. Baseline Characteristics of Hospitalized Children Aged <18 Years With Infection, January 2015 to March 2023
eTable 3. Sensitivity Analysis With Estimated Fraction of Henoch-Schönlein Purpura Potentially Attributable to SARS-CoV-2
eFigure 1. Seasonal Pattern of the Incidence of HSP and SARS-CoV-2 Infections (N=41,484) per 100,000 Children Aged <18 Years in France
eFigure 2. Correlograms and Residuals Analysis of the Final Quasi-Poisson Regression Model for the Estimated Fraction of ACS Attributable to Seasonal Pathogens
eTable 4. Sensitivity Analyses for the Estimated Fraction of HSP Potentially Attributable to Seasonal Pathogens
eMethods. Details of the Quasi-Poisson Regression Model

This supplementary material has been provided by the authors to give readers additional information about their work.

© 2024 Felix A et al. *JAMA Network Open.*

eTable 1. *ICD-10* Codes for Seasonal Pathogens in the General Population

Diagnosis	*ICD-10* code	Label
**RSV**	J121	Respiratory syncytial virus pneumonia
	J205	Acute bronchitis due to respiratory syncytial virus
	J210	Acute bronchiolitis due to respiratory syncytial virus
**Influenza virus**	J10	Influenza due to other identified influenza virus
	J100	Influenza due to other identified influenza virus with pneumonia
	J101	Influenza due to other identified influenza virus with other respiratory manifestations
	J108	Influenza due to other identified influenza virus with other manifestations
**hPIV**	J122	Parainfluenza virus pneumonia
	J204	Acute bronchitis due to parainfluenza virus
**hMPV**	J123	Human metapneumovirus pneumonia
	J211	Acute bronchiolitis due to human metapneumovirus
**hAdV**	J120	Adenoviral pneumonia
	A082	Adenoviral enteritis
	A851	Adenoviral encephalitis
	B340	Adenovirus infection, unspecified
**hRV/hEV**	J206	Acute bronchitis due to rhinovirus
	A850	Enteroviral encephalitis
	A870	Enteroviral meningitis
	A880	Enteroviral exanthematous fever
	B341	Enterovirus infection, unspecified
	B971	Unspecified enterovirus as the cause of diseases classified elsewhere
**hCoV**	B972	Coronavirus as the cause of diseases classified elsewhere
	B342	Coronavirus infection, unspecified
**Rotavirus**	A080	Rotaviral enteritis
**Norovirus**	A081	Acute gastroenteropathy due to Norwalk agent
**VZV**	B01	Varicella
	B010	Varicella meningitis
	B011	Varicella encephalitis and encephalomyelitis
	B012	Varicella pneumonia
	B018	Varicella keratitis
	B019	Varicella without complication
**SARS-CoV-2**	U071	Covid-19
S. pneumoniae	A403	Sepsis due to *Streptococcus pneumoniae*
	G001	Pneumococcal meningitis
	J13	Pneumonia due to *Streptococcus pneumoniae*
H. influenzae	J14	Pneumonia due to *Hemophilus influenzae*
M. pneumoniae	J157	Pneumonia due to *Mycoplasma pneumoniae*
	A493	Mycoplasma infection, unspecified site
C. pneumoniae	J160	Chlamydial pneumonia

Abbreviations: RVs/EVs, human rhinovirus-enterovirus; RSV, respiratory syncytial virus; hPIV, human parainfluenza virus; hMPV, human metapneumovirus; hAdV, human adenovirus; hCoV, human coronavirus; VZV, varicella zoster virus; SARS-CoV-2, severe acute respiratory syndrome coronavirus 2; Covid-19, coronavirus disease 2019.

© 2024 Felix A et al. *JAMA Network Open.*

eTable 2. Baseline Characteristics of Hospitalized Children Aged <18 Years With Infection, January 2015 to March 2023

Pathogen	No. of cases, No. (%)	Age, median (IQR), years	Duration of stay, median (IQR), days	Death, No. (%)
**hRV/EV**	292,434	0.9 (0.2-3)	6 (3-15)	4211 (1.4)
**Influenza virus**	56,495	2 (0.8-5)	2 (1-3)	173 (0.31)
**RSV**	194,613	0.24 (0.1-0.5)	4 (2-6)	155 (0.08)
**hPIV**	2073	1 (0.4-3)	4 (2-8)	31 (1.5)
**hMPV**	8181	0.5 (0.2-1)	4 (2-7)	42 (0.51)
**hAdV**	29,737	1 (0.5-2)	3 (2-5)	125 (0.42)
**hCoV**	5519	0.8 (0.2-3)	3 (2-6)	34 (0.62)
**VZV**	21,796	2 (0.97-3)	2 (1-4)	29 (0.13)
**Norovirus**	5777	1 (0.5-2)	3 (1-5)	16 (0.28)
**Rotavirus**	113,664	1 (0.6-2)	2 (2-3)	73 (0.06)
S. pneumoniae	9037	2 (0.9-5)	4 (2-9)	145 (1.6)
S. pyogenes	8576	4 (2-8)	3 (1-6)	54 (0.63)
M. pneumoniae	8757	5 (3-9)	3 (2-5)	12 (0.14)
C. pneumoniae	451	8 (2-15)	4 (2-7)	2 (0.44)

Median age and duration of stay are presented as medians (IQR), and categorical data (number of cases, death) as numbers (%).
Abbreviations: hRV/EV, human rhinovirus-enterovirus; RSV, respiratory syncytial virus; hPIV, human parainfluenza virus; hMPV, human metapneumovirus; hAdV, human adenovirus; hCoV, human coronavirus; VZV, varicella zoster virus.

© 2024 Felix A et al. *JAMA Network Open.*

eTable 3. Sensitivity Analysis With Estimated Fraction of Henoch-Schönlein Purpura Potentially Attributable to SARS-CoV-2

	Estimated fraction of HSP [a]	
**Pathogen**	**% (95% CI)**	***P* value**
SARS-CoV-2	-2.9 (-6.5 to 0.61)	.11

[a] Analysis by seasonally-adjusted quasi-Poisson regression model.
Abbreviations: SARS-CoV-2, severe acute respiratory syndrome coronavirus 2.

© 2024 Felix A et al. *JAMA Network Open.*

eFigure 1. Seasonal Pattern of the Incidence of HSP and SARS-CoV-2 Infections (N=41,484) per 100,000 Children Aged <18 Years in France

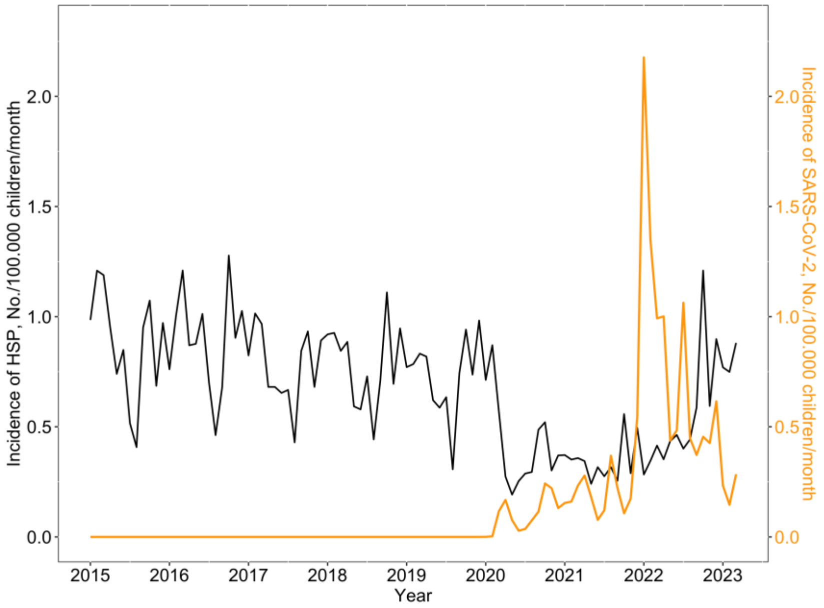

The black line shows the monthly incidence of HSP per 100,000 children. The orange line shows the monthly incidence of SARS-CoV-2 infections per 100,000 children.

© 2024 Felix A et al. *JAMA Network Open.*

eFigure 2. Correlograms and Residuals Analysis of the Final Quasi-Poisson Regression Model for the Estimated Fraction of ACS Attributable to Seasonal Pathogens

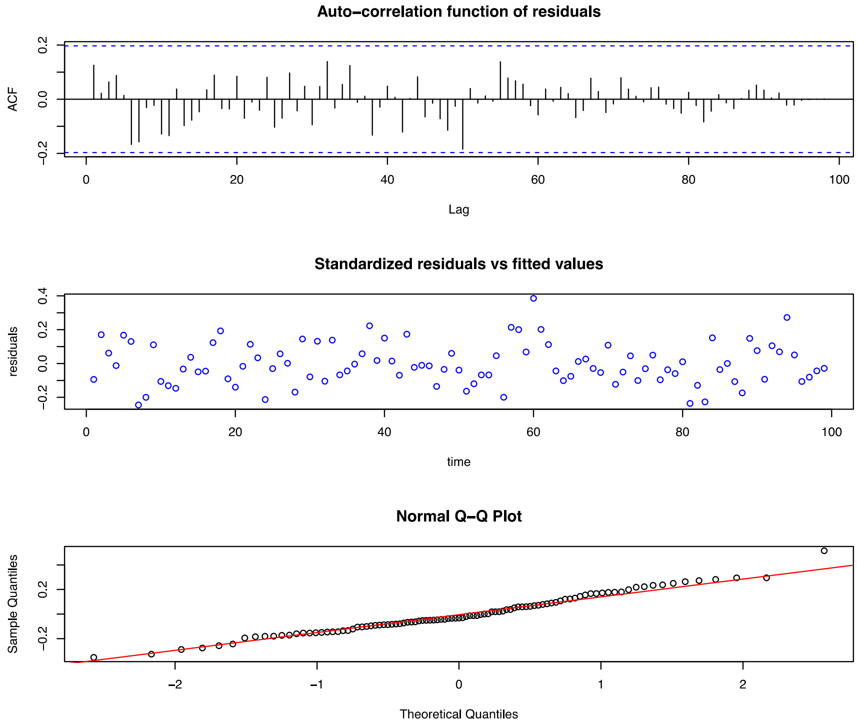

To assess the quality of the Quasi-Poisson model, we used correlograms (autocorrelation and partial autocorrelation functions which measure the linear relationship between lagged values of a time series) and residuals analysis. Inspection of the correlograms relies on identifying remaining autocorrelation or seasonal pattern of the residuals. The significance of any remaining autocorrelation or seasonality is defined by a correlation higher than +1.96 standard error or lower than -1.96 standard error for each lag of the time series. We checked whether the residuals of the models were normally distributed and had a constant variance over time. The correlograms were satisfactory (no remaining autocorrelation nor seasonal pattern of the residuals).
ACF, autocorrelation function.

© 2024 Felix A et al. *JAMA Network Open.*

eTable 4. Sensitivity Analyses for the Estimated Fraction of HSP Potentially Attributable to Seasonal Pathogens

	Estimated fraction of HSP					
	Model without highly correlated covariates[a,b]		Model with monthly counts of cases[a]		Model with 6- and 12-month periods seasonality[a]	
**Pathogen**	**% (95% CI)**	***P* value**	**% (95% CI)**	***P* value**	**% (95% CI)**	***P* value**
**Virus**						
RVs/EVs	.		17.3 (4.0 to 30.6)	.01	17.8 (4.6 to 31.0)	.009
Influenza virus	0.74 (-3.1 to 4.6)	.71	0.59 (-3.6 to 4.7)	.78	0.28 (-4.0 to 4.5)	.90
RSV	.		-6.9 (-14.4 to 0.60)	.08	-5.6 (-12.9 to 1.7)	.14
hPIV	10.3 (3.3 to 17.4)	.005	8.1 (-0.92 to 17.1)	.08	6.6 (-2.5 to 15.8)	.16
hMPV	-11.0 (-18.5 to -3.5)	.005	-7.3 (-15.6 to 1.1)	.09	-6.8 (-15.3 to 1.7)	.12
hAdV	.		-7.8 (-27.0 to 11.5)	.43	-8.8 (-28.5 to 10.9)	.38
hCoV	.		1.8 (-7.9 to 11.5)	.72	2.1 (-7.6 to 11.9)	.67
VZV	3.0 (-9.6 to 15.6)	.64	-2.6 (-17.2 to 12.0)	.73	-3.1 (-17.9 to 11.8)	.69
Norovirus	0.03 (-8.6 to 8.7)	.99	-2.9 (-11.6 to 5.9)	.52	-3.0 (-12.0 to 6.0)	.52
Rotavirus	.		5.9 (-2.7 to 14.5)	.18	6.9 (-1.6 to 15.5)	.12
**Bacteria**						
S. pneumoniae	35.2 (21.9 to 48.6)	<.001	37.2 (22.2 to 52.1)	<.001	37.7 (23.0 to 52.4)	<.001
S. pyogenes	27.6 (20.0 to 35.3)	<.001	25.8 (16.9 to 34.7)	<.001	25.9 (17.0 to 34.8)	<.001
M. pneumoniae	1.5 (-10.4 to 13.4)	.81	7.6 (-4.9 to 20.2)	.24	8.8 (-3.8 to 21.4)	.17
C. pneumoniae	1.5 (-7.5 to 10.6)	.74	1.0 (-8.3 to 10.3)	.83	1.4 (-8.0 to 10.8)	.77

[a] Analysis by seasonally-adjusted quasi-Poisson regression model
[b] Model excluding the following highly correlated covariates (RVs/EVs, RSV, hAdV, hCoV, rotavirus)
Abbreviations: HSP, Henoch-Schönlein purpura; RVs/EVs, rhinovirus/enterovirus; RSV, respiratory syncytial virus; hPIV, human parainfluenza virus; hMPV, human metapneumovirus; hAdV, human adenovirus; hCoV, human coronavirus; VZV, varicella zoster virus.

© 2024 Felix A et al. *JAMA Network Open.*

eMethods. Details of the Quasi-Poisson Regression Model

First part: To estimate the changes in HSP incidence associated with the implementation and relaxation of NPIs.

The quasi-Poisson interrupted time series regression model can be written as:

Log(Yt) = $\beta_0 + \beta_1$ * time$_t$ + β_2 * NPI implementation + β_3 * Time after NPI lifting + β_4 * cos(θ) + β_5 * sin(θ) + β_6 * cos(ϕ) + β_7 * sin(ϕ) +ϵ_t **(Equation 1)**

Yt: monthly rate (per 100,000 children) hospitalized for HSP in France. Yt is in a logarithmic base, therefore model's estimates need to be exponentiated to be interpreted as mean percentage changes.

exp(β_0): estimate of the baseline level (rate per 100,000 children and adolescents) in January 1, 2015

exp(β_1): estimate of the slope (trend) in the pre-NPI period.

Time$_t$**:** time elapsed since the beginning of the study. A continuous variable measured in months ranging from January 1, 2015 to March 31, 2023.

exp (β_2): estimate of the change in level after the NPI implementation.

NPI implementation: a categorical variable coded 0 before March 2020 coded 1 afterwards.

exp(β_3): estimate of change in slope after the NPI lifting

Time after NPI lifting: a continuous variable counting the number of months from April 1, 2021 to March 31, 2023.

exp(β_4): estimate of the harmonic functions for fitting a harmonic trend (cosine-sine trend, Fourier regression) for 12-month period

exp(β_5): estimate of the harmonic functions for fitting a harmonic trend (cosine-sine trend, Fourier regression) for 12 month period

θ: 2πkt. t is the frequency of the function ($\frac{1}{period}$), k is the number of sine and cosine pairs (k = 1 for 12-month period)

The sine and cosine terms fit a seasonal baseline using annual (12 months, $\theta = \frac{2*\pi*1}{12}$) period.

exp(β_6): estimate of the harmonic functions for fitting a harmonic trend (cosine-sine trend, Fourier regression) for semi-annual (6-month) period

exp(β_7): estimate of the harmonic functions for fitting a harmonic trend (cosine-sine trend, Fourier regression) for semi-annual (6-month) period

ϕ: 2πkt. t is the frequency of the function ($\frac{1}{period}$), k is the number of sine and cosine pairs (k = 2 for semi -annual period)

The sine and cosine terms fit a seasonal baseline using semi-annual (6 months, $\phi = \frac{2*\pi*2}{12}$) period.

ϵ_t : the model's residual error

Second part: To estimate the fraction of HSP attributable to each pathogen of interest:

Log(Yt) = $\beta_0 + \beta_1$ * time$_t$ + β_2 * NPI implementation + β_3 * Time after NPI lifting + β_4 * cos(θ) + β_5 * sin(θ) + β_6 * cos(ϕ) + β_7 * sin(ϕ) + β_8 * cos(ω) + β_9 * sin(ω) + β_{10} * Human rhino-enterovirus + β_{11} * Influenza + β_{12} * RSV + β_{13} * Human parainfluenza virus + β_{14} * Human metapneumovirus + β_{15} * Human adenovirus + β_{16} * Non SARS-CoV-2 human coronavirus + β_{17} * Varicella zoster virus + β_{18} * Norovirus + β_{19} * Rotavirus + β_{20} * *Streptococcus pneumonia* + β_{21} * *Streptococcus pyogenes* + β_{22} * *Mycoplasma pneumoniae* + β_{23} * *Chlamydia pneumoniae* + ϵ_t **(Equation 2)**

Yt: monthly incidence rate (per 100,000 children) hospitalized for HSP in France. Yt is in a logarithmic base, therefore model's estimates need to be exponentiated to be interpreted as mean percentage changes.

exp(β_0): estimate of the baseline level (rate per 100,000 children and adolescents) in January 1, 2015

exp(β_1): estimate of the slope (trend) in the pre-NPI period.

Time$_t$**:** time elapsed since the beginning of the study. A continuous variable measured in months ranging from January 1, 2015 to March 31, 2023.

exp (β_2): estimate of the change in level after the NPI implementation.

NPI implementation: a categorical variable coded 0 before March 2020 coded 1 afterwards.

© 2024 Felix A et al. *JAMA Network Open.*

$\exp(\beta_3)$: estimate of change in slope after the NPI lifting
Time after NPI lifting: a continuous variable counting the number of months from April 1, 2021 to March 31, 2023.

$\exp(\beta_4)$: estimate of the harmonic functions for fitting a harmonic trend (cosine-sine trend, Fourier regression) for 12 month period
$\exp(\beta_5)$: estimate of the harmonic functions for fitting a harmonic trend (cosine-sine trend, Fourier regression) for 12 month period
θ: 2πkt. t is the frequency of the function ($\frac{1}{period}$), k is the number of sine and cosine pairs (k = 1 for 12 month period)
The sine and cosine terms fit a seasonal baseline using annual (12 months, $\theta = \frac{2*\pi*1}{12}$) period.

$\exp(\beta_6)$: estimate of the harmonic functions for fitting a harmonic trend (cosine-sine trend, Fourier regression) for semi-annual (6 month) period
$\exp(\beta_7)$: estimate of the harmonic functions for fitting a harmonic trend (cosine-sine trend, Fourier regression) for semi-annual (6 month) period
ϕ: 2πkt. t is the frequency of the function ($\frac{1}{period}$), k is the number of sine and cosine pairs (k = 2 for semi -annual period)
The sine and cosine terms fit a seasonal baseline using semi-annual (6 months, $\phi = \frac{2*\pi*2}{12}$) period.

$\exp(\beta_8)$: estimate of the harmonic functions for fitting a harmonic trend (cosine-sine trend, Fourier regression) for trimestral (3 month) period
$\exp(\beta_9)$: estimate of the harmonic functions for fitting a harmonic trend (cosine-sine trend, Fourier regression) for trimestral (2 month) period
ω: 2πkt. t is the frequency of the function ($\frac{1}{period}$), k is the number of sine and cosine pairs (k = 3 for trimestral period)
The sine and cosine terms fit a seasonal baseline using trimestral (3 months, $\omega = \frac{2*\pi*3}{12}$) period.

Human rhino-enterovirus: monthly incidence rate (per 100,000 children) hospitalized for human rhino-enterovirus infection in France.
Influenza: monthly incidence rate (per 100,000 children) hospitalized for flu in France.
RSV: monthly incidence rate (per 100,000 children) hospitalized for RSV infection in France.
Human parainfluenza virus: monthly incidence rate (per 100,000 children) hospitalized for human parainfluenza virus infection in France.
Human metapneumovirus: monthly incidence rate (per 100,000 children) hospitalized for human metapneumovirus infection in France.
Human adenovirus: monthly incidence rate (per 100,000 children) hospitalized for human adenovirus infection in France.
Non-SARS-CoV-2 human coronavirus: monthly incidence rate (per 100,000 children) hospitalized for non-SARS-CoV-2 human coronavirus infection in France.
Varicella zoster virus: monthly incidence rate (per 100,000 children) hospitalized for varicella zoster virus in France.
Norovirus: monthly incidence rate (per 100,000 children) hospitalized for norovirus infection in France.
Rotavirus: monthly incidence rate (per 100,000 children) hospitalized for rotavirus infection in France.
Streptococcus pneumonia: monthly incidence rate (per 100,000 children) hospitalized for pneumococcal infection in France.
Streptococcus pyogenes: monthly incidence rate (per 100,000 children) hospitalized for *streptococcus group A infection* in France.
Mycoplasma pneumoniae: monthly incidence rate (per 100,000 children) hospitalized for *Mycoplasma pneumoniae* infection in France.
Chlamydia pneumoniae: monthly incidence rate (per 100,000 children) hospitalized for *Chlamydia pneumoniae* infection in France.

ϵ_t: the model's residual error

© 2024 Felix A et al. *JAMA Network Open.*

The fraction of HSP hospitalizations attributable to each pathogen in circulation
Estimated incidence of HSP: hospitalization rates estimated by **equation 2**
Expected incidence of HSP: hospitalization rates forecast by **equation 2** by setting each pathogen's incidence to zero (i.e. the expected incidence rate of HSP if the corresponding pathogen was not in circulation)
During the total study period

$$FA = \left(\frac{\text{Estimated incidence of HSP} - \text{Expected incidence of HSP}}{\textit{Estimated}\ \text{incidence of HSP}}\right) * 100$$

© 2024 Felix A et al. *JAMA Network Open.*
