# Supplementary material for: Common Seasonal Pathogens and Epidemiology of Henoch-Schönlein Purpura Among Children
Source: JAMA Netw Open. 2024 Apr 5;7(4):e245362. doi: 10.1001/jamanetworkopen.2024.5362 (PMC10998156; doi:10.1001/jamanetworkopen.2024.5362)
Supplement: Supplement 2. — Data Sharing Statement [file jamanetwopen-e245362-s002.pdf]

Data Sharing Statement

Felix. Common Seasonal Pathogens and Epidemiology of Henoch-Schönlein Purpura Among Children. *JAMA Netw Open*. Published April 05, 2024.
doi:10.1001/jamanetworkopen.2024.5362

Data
Data available: Yes
Data types: Deidentified participant data
How to access data: Individual participant data required to reach aims in an approved proposal, after de-identification, will be made available to investigators whose proposed use of the data has been approved by the study's Executive Committee. Proposals may be submitted up to 36 months after article publication and should be directed to ulrich.meinzer@aphp.fr.
When available: With publication

Supporting Documents
Document types: None

Additional Information
Who can access the data: Individual participant data required to reach aims in an approved proposal, after de-identification, will be made available to investigators whose proposed use of the data has been approved by the study's Executive Committee. Proposals may be submitted up to 36 months after article publication and should be directed to ulrich.meinzer@aphp.fr.
Types of analyses: For non-commercial purposes
Mechanisms of data availability: Individual participant data required to reach aims in an approved proposal, after de-identification, will be made available to investigators whose proposed use of the data has been approved by the study's Executive Committee. Proposals may be submitted up to 36 months after article publication and should be directed to ulrich.meinzer@aphp.fr.
Any additional restrictions: .
